# The Status of Digital Dental Technology Implementation in the Saudi Dental Schools’ Curriculum: A National Cross-Sectional Survey for Healthcare Digitization

**DOI:** 10.3390/ijerph20010321

**Published:** 2022-12-25

**Authors:** Hayam A. Alfallaj, Kelvin I. Afrashtehfar, Ali K. Asiri, Farah S. Almasoud, Ghaida H. Alnaqa, Nadia S. Al-Angari

**Affiliations:** 1 Department of Restorative and Prosthetic Dental Sciences, College of Dentistry, King Saud bin Abdulaziz University for Health Sciences, Riyadh P.O. Box 3660, Saudi Arabia; 2 King Abdullah International Medical Research Center, Riyadh P.O. Box 3660, Saudi Arabia; 3 Evidence-Based Practice Unit, Clinical Sciences Department, College of Dentistry, Ajman University, Ajman P.O. Box 346, United Arab Emirates; 4 Department of Reconstructive Dentistry and Gerodontology, School of Dental Medicine, University of Bern, 3010 Bern, Switzerland; 5 Dental Department, King Abdulaziz Medical City, Ministry of National Guard Health Affairs, Riyadh P.O. Box 22490, Saudi Arabia; 6 College of Dentistry, King Saud bin Abdulaziz University for Health Sciences, Riyadh P.O. Box 3660, Saudi Arabia

**Keywords:** dental education, digital dentistry, CAD-CAM, intraoral scanner, dental technology, curriculum

## Abstract

*Objective:* The primary objective of this cross-sectional national study was to investigate the status of digital dental technology (DDT) adoption in Saudi Arabian undergraduate dental education. A secondary objective was to explore the impact of dental schools’ funding sources to incorporate digital technologies. *Methods:* A self-administered questionnaire was distributed to the chairpersons of prosthetic sciences departments of the 27 dental schools in Saudi Arabia. If any department chairman failed to respond to the survey, a designated full-time faculty member was contacted to fill out the form. The participants were asked about the school’s sector, DDT implementation in the curriculum, implemented level, their perceptions of the facilitators and challenges for incorporating DDT. *Results:* Of the 27 dental schools (18 public and 8 private), 26 responded to the questionnaire (response rate: 96.3%). The geographic distribution of the respondent schools was as follows: 12 schools in the central region, 6 in the western region, and 8 in other regions. Seventeen schools secure and preserve patients’ records using electronic software, whereas nine schools use paper charts. Seventeen schools (64,4%) implemented DDT in their curricula. The schools that did not incorporate DDT into their undergraduate curricula were due to not being included in the curriculum (78%), lack of expertise (66%), untrained faculty and staff (44%), and cost (33%). *Conclusions:* This national study showed that digital components still need to be integrated into Saudi Arabian dental schools’ curricula and patient care treatment. Additionally, there was no association between funding sources and the DDT implementation into the current curricula. Consequently, Saudi dental schools must emphasize the implementation and utilization of DDT to align with Saudi Vision 2030 for healthcare digitization and to graduate competent dentists in digital dental care.

## 1. Introduction

Incorporating digital technologies, such as computer-aided design (CAD) and computer-aided manufacturing (CAM), in dental practice has exponentially grown over the last years, not only being an alternative to traditional dental techniques but also replacing them [1,2]. For example, the use of intraoral scanners (IoS), designing final restorations using computer software (i.e., CAD), fabricating single or multiple tooth restorations (i.e., CAM), and fabricating partial (PRDPs) or complete removable dental prostheses (CRDPs) using subtractive (s-CAM) or additive manufacturing technology (a-CAM) [3].

Moreover, digital technology has also been used in implant dentistry [4,5,6,7]. A restorative dentist can implement a partial or fully digital workflow by precisely planning the implants’ positions, manufacturing surgical guides, and scanning the implant in the patient’s mouth to fabricate final prostheses. Other digital applications include using software that aids in digital smile design (DSD), in which a digital wax-up can be used to generate a virtual cast and, consequently, printing an index to be used for direct clinical mock-up [8,9,10,11,12]. In addition, the patient may immediately visualize the treatment results based on a digital representation of the treatment outcome and approve or suggest changes, offering them a greater understanding of the course of treatment [13]. Furthermore, using digital technology in the maxillofacial prosthodontics subspecialty is crucial to making impressions. The complexity of the orofacial disease and the patient’s clinical situation mandates the digital approach. So, these advances in digital technology will aid in the patient’s experience and produce an accurate maxillofacial prosthesis with less time and visits and a more favorable outcome [13,14]. The convenience of using advanced technology in dentistry has not only improved the quality of the treatment but also facilitated the communication between restorative dentists and lab technicians, improved patients’ acceptance of the proposed treatment, and simplified interdisciplinary treatment planning [15,16].

Besides the advantages of integrating different digital technologies in routine dental practice, their use has proven beneficial as an educational tool, especially at the undergraduate (UG) level. For example, in a study by Matthisson et al., the use of visual feedback from IoSs combined with the Prep-check software (Sirona) resulted in a better agreement between students’ self-assessment and instructors’ assessment of a crown preparation in a preclinical training setting [17]. In addition, other studies have shown that using IoSs significantly reduced the evaluation subjectivity and improved inter- and intra-examiner agreement when evaluating crown preparation in a preclinical lab training setting [18,19].

As technology continues to develop and integrate into daily dental practice, dental students shall be adequately trained or at least be exposed to such devices and workflows before graduating from dental schools to be competent in providing contemporary dental treatments [20,21,22]. The use of dental technology in UG dental school has been a primary matter of research in dental education in the last few years. For example, a study in the United Kingdom (UK) reported that 55% of dental schools did not teach digital dental technology in their Bachelor of Dental Surgery (BDS) curriculum [23]. The authors also noted that half of the schools that teach digital technology limit their teaching to the didactic part [23]. While in North America, a recent survey reported that over 90% of dental schools use CAD/CAM technology in the UG curriculum, among which 55% reported using IoSs exclusively in clinical training [15]. Another study reported that incorporating CAD/CAM technology for indirect coronal restoration was more frequent in pre-clinical didactic components (76%) [24]. Over half of the schools reported using CAD/CAM in providing clinical patient care. The least taught modern technology in didactic, practical, or clinical components was digital CRDPs, commonly known as digital dentures [24]. Likewise, a study reported that only 12% of the US dental schools incorporated digital denture fabrication in the UG curriculum [25].

The Saudi Vision 2030 emphasizes digital healthcare transformation; thus, this study aimed to explore the status of DDT adoption and teaching in Saudi Arabian undergraduate dental education. A secondary objective was to explore the impact of dental schools’ funding sources on incorporating various digital technologies into their undergraduate curriculum.

## 2. Materials and Methods

A national prospective cross-sectional survey for healthcare digitization was conducted to analyze the extent of the use of digital technologies in dental schools and to investigate if the schools’ funding source might influence the integration of digital technologies into their UG curricula. The research team developed the questionnaire, and some of the questions were adapted from Brownstein et al. and Catham et al. studies [23,24]. The self-administered web-based survey (SurveyMonkey) consisted of 21 questions that were directed to the Department of Prosthodontics chairpersons of the 27 dental schools in Saudi Arabia. Hence, the sampling method was a voluntary response sample. The survey was validated through experts’ opinions (2 full-time prosthodontic faculty members). Then, it was distributed in September and October 2021 through the official email addresses of department chairpersons obtained from the schools’ websites. The survey link was embedded within an email. After two weeks, non-respondents received follow-up emails. If the chairperson of the corresponding school did not respond in one month, a full-time prosthodontic faculty member, who had access to the required information, was contacted instead. No duplicate responses could be received from the same school, as only one faculty was contacted at the time. Each school was designated a randomly generated identification code known only to the study’s primary investigator to secure anonymity. Institutional review board (IRB) approval was obtained from King Abdullah International Medical Research Center (SP20/504/R).

The participants were asked about their school’s characteristics, such as funding source, geographic region, and method of saving patients’ records, whether electronic or paper charts. Respondents who reported DDT incorporation were asked to indicate the type of technology (i.e., 2D digital radiography, cone beam technology, rotary endodontics, chairside milling, digitally designed stents for implant placement, IoS, soft- or hard-tissue lasers, extra-oral scanning, digital dentures). Additionally, the participants were asked at which level in their curricula DDTs were being taught (i.e., pre-clinical didactic, pre-clinical laboratory, clinical didactic, or clinical patient experience). Respondents that reported a lack of DDT implementation were asked about the reasons behind not including DDTs and whether they intend to implement DDTs in their curricula in the future or not.

In addition, participants were asked to indicate their level of satisfaction with the implemented DDTs at their schools by adding a mark on a 100 mm visual analog scale (where 100 indicated fully satisfied and 0 indicated entirely dissatisfied). The satisfaction value was determined by measuring the distance from the left end of the scale to the mark in millimeters and then expressed as a percentage.

### Statistical Analysis

Data analysis was performed using SPSS Statistics (version 21, IBM, Armonk, NY, USA). Frequencies and percentages were calculated for the categorical variables. Tables and box plots were used for better demonstration and easy visualization. Chi-square tests were used for statistical analyses. All the tests were considered significant if the *p*-value was less than 0.05.

## 3. Results

Out of the 27 dental schools (18 public and 8 private) in Saudi Arabia, 26 responded to this national questionnaire, with a response rate of 96.3%. The geographic distribution of the responded schools was as follows: 12 colleges in the central region, 6 in the western region, 2 in the eastern region, 2 in the northern region, and 4 in the southern region. Moreover, 17 (15 public and 2 private) out of 26 respondent schools (65.4%) saved patient records using electronic software, whereas 9 schools (3 public and 6 private) used paper charts (34.6%). A total of 17 (65.4%) schools (12 public and 5 private) implemented DDTs in their curricula (Table 1). Results showed that most dental schools implement DDTs by combining didactic, laboratory, and clinical practice (Table 1). The chi-square test showed no statistically significant association (*p* = 0.837) in the DDTs adoption among dental schools based on their funding sources.

Furthermore, 9 (34.6%) dental schools (6 public and 3 private) reported the lack of incorporation of DDTs into their UG curriculum for the following reasons: cost (33%), untrained faculty (44%), lack of technical expertise (66%), and not being part of the curriculum (78%). Out of them, 5 colleges planned to incorporate DDTs within 1–2 years, 2 within 3–5 years, 1 is still in the discussion phase, and 1 school needs to plan it. Although 17 colleges incorporated DDTs in their curricula, only 6 use IoS (2 Planmeca Emerald, 1 Cerec OmniCam, 1 3shape Trios, 1 Carestream CS, and 1 3M True Definition). The most common prosthesis students fabricate using CAD/CAM is single crowns. Moreover, only 3 colleges required students to fabricate same-day chairside CAD/CAM restorations. Remarkably, most schools’ laboratories 87.5% are using DDTs.

**Figure 1:**
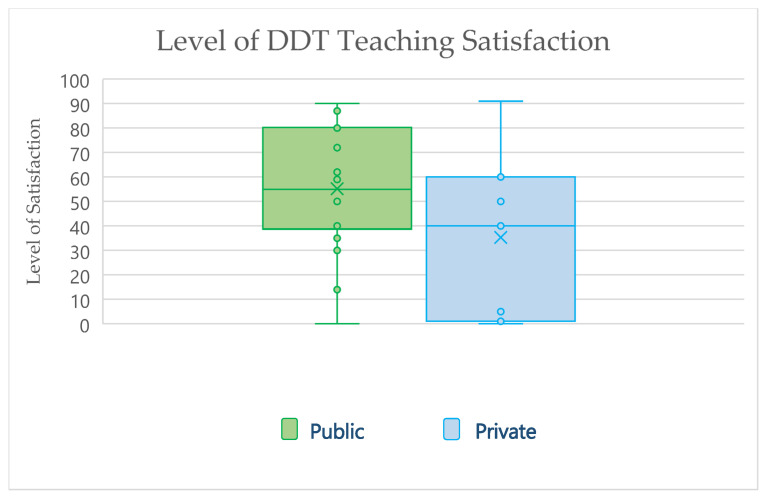
Responses of Saudi Arabia dental schools reporting their level of satisfaction in teaching digital dental technologies in their curricula (18 public; 7 private). One of the included private schools failed to complete the satisfaction level. DDT, digital dental technology.

**Figure 2:**
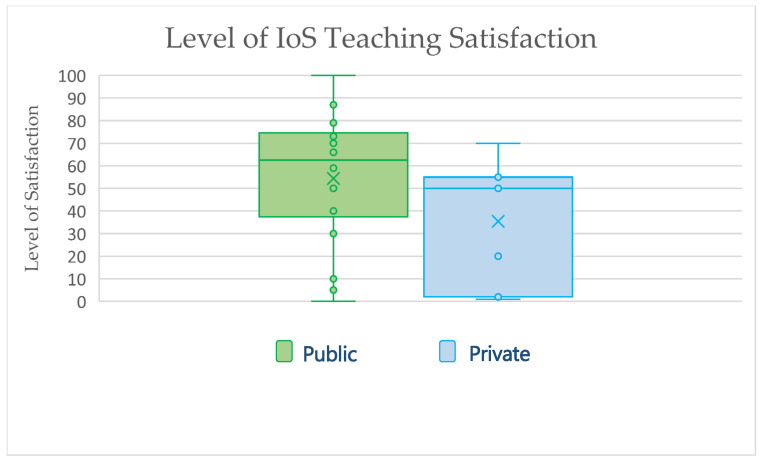
Responses of Saudi Arabia dental schools reporting their level of satisfaction in teaching intraoral scanning in their curricula. One of the included private schools failed to complete the satisfaction level. IoS, intraoral scanner.

The respondents’ average satisfaction level regarding DDT involvement in their schools’ curricula was 55% and 35% for public and private schools, respectively (Figure 1). When the participants were asked about IoS teaching and utilization in their schools, the responses were 54% and 35% for public and private schools, respectively (Figure 2).

## 4. Discussion

While the demand to integrate new digital technologies into dental schools’ curricula is essential to graduate competent dentists who can provide contemporary patient care, the task may be challenging and require more work for dental school authorities [26,27,28,29]. For instance, the school’s administrators need to justify the grants required to acquire the necessary technologies. In addition, faculty members and clinical instructors must update their courses and keep up with the newest technologies. These factors and others may negatively affect the decision of dental schools to incorporate newer digital technologies and modify their existing curricula.

While the findings of this study depicted a general deficiency in integrating the new digital technologies into dental schools’ curricula, there is a clear tendency toward incorporating dental technologies more in the didactic component of their curriculum rather than in practical or clinical patient care. This could be explained by the simplicity of modifying the content of the didactic component without the need for administrative approval, additional fund, or technical skills necessary to acquire and operate the new devices and software. This study also showed that the most significant exposure of Saudi dental schools’ students to the available DDTs was in the clinical didactic courses. In contrast, US dental students have more exposure to the technologies in the pre-clinical didactic courses [24]. However, the underutilization of the advanced digital technologies acquired by some Saudi schools can be caused by untrained faculty members who need to learn how to operate the existing resources. Thus, a sensible recommendation is that schools ensure their dental faculty are trained to facilitate the adoption of DDT instruction to their students.

In the current study, DDTs are implemented in 17 (65.4%) Saudi Arabian dental schools’ curricula with unsignificant association amongst dental schools based on their funding source, whether a governmental or private institution. Moreover, nine schools (34.6%) reported a lack of DDTs implementation in their curriculum, of which three were private schools. Given that the cost of education at these schools is high, it is the responsibility of the schools to ensure adequate exposure of their students to the available technologies to improve their future practice and patient care.

The most-implemented DDTs in Saudi schools are 2D digital radiography and cone-beam (CBCT) technology, while the least-implemented DDTs were digital dentures. The findings of this study are consistent with Brownstein et al. [24], who reported that digital radiography is the most often implemented DDTs in US dental schools, and digital dentures were the least implemented DDTs [24]. Similar to the results published by Ishida et al. [30], the involvement of the digital complete dentures component in the pre-clinical training was less frequent than the didactic and clinical patients care, with only 7.7% of Saudi schools using digital dentures in clinical patient care in comparison to 37.5% US dental schools.

Although some schools incorporated DDTs more than others, 34.6% of Saudi dental schools reported a lack of integration of DDTs in their curricula. However, a direct comparison to the result published by Chatham et al., which stated that 45% of schools in the UK did not incorporate DDTs, may be an injustice as more advanced technologies are expected to be incorporated in the UK dental schools since their publication [23]. Likewise, comparing percentages of the dental schools that implemented digital technologies among different nations must be interpreted carefully, as the response rates among the reported study are highly variable [23,24,25,30].

Besides the benefits of digital technologies that directly help patient care, this study also investigated using electronic health records to preserve patient data in Saudi Arabian dental schools. The study data showed that 17 (95.4%) schools used computer software to maintain patients’ records, while 9 (34.6%) schools used paper charts. Electronic health records can efficiently improve data management and simplify data extraction for researchers. There is still some resistance to using electronic software for cost reasons, privacy concerns, and staff resistance [31].

The study participants’ satisfaction levels regarding Incorporating digital technologies into their curricula were diverse. While the average satisfaction values were 55% and 35% for public and private schools, the box plot diagram showed a broad level of satisfaction among participants, with public schools representative tending to show more satisfaction (Figure 1). A similar pattern of satisfaction was noticed when the respondents were asked about teaching and utilizing IoS in the UG curricula, with more satisfaction observed among public school respondents (Figure 2). The lower level of satisfaction observed by the private school’s respondents could be explained by the high pressure facing private schools’ authorities to adopt new technologies into their curricula to graduate competent dentists.

The presented study is considered the first to investigate the status of DDTs involvement in UG curricula, with a high response rate exceeding 96% of participation. However, the results are limited to the information provided by the department chairperson or prosthodontist full-time faculty members, which may reduce the precision of answering all questionnaire components. Moreover, despite the survey containing similar questions from previous British and North American studies using validated questionnaires, the current survey validity was based on the perspectives of the research team and two field experts. Additionally, the broad spectrum of the questions in the distributed survey might also be considered a limitation of this study.

Therefore, future narrow-focusing, reliable, validated studies that investigate the implementation of certain technology might give a better understanding of the current UG curricula. Moreover, follow-up survey studies are recommended to observe the future integration of digital technologies into Saudi dental schools’ curricula and to compare it to other countries with leading dental schools. Additionally, a representative sample of students from each dental school can participate in a survey to determine the effectiveness of digital dentistry in the curriculum and assess student satisfaction and confidence in undertaking a digital workflow.

## 5. Conclusions

The findings from this national online survey showed that Saudi Arabian dental schools still need to focus their efforts on accelerating the implementation of digital dentistry technologies in their curricula to satisfy the laboratory and clinical training components. Only 65.4% of Saudi Arabia dental schools reported integrating digital technologies in the undergraduate curricula, with no association between funding sources and the implementation of DDTs. Therefore, it is recommended that Saudi dental schools increase the adoption and utilization of DDTs. In turn, schools will graduate competent dentists in digital dental care, which aligns with the Saudi Vision 2030 for healthcare digitization.

## Figures and Tables

**Table 1:** Responses of 17 dental schools that implemented dental dentistry into their curriculum *.

	Preclinical Didactic	Preclinical Laboratory	Clinical Didactic	Clinical Patient Experience	No. of Schools
**2D digital radiography**	11 (68.75%) (8 public/3 private)	8 (50%) (6 public/2 private)	13 (81.25%) (10 public/3 private)	11 (68.75%) (9 public/2 private)	16 (12 public/4 private)
**3D digital radiography (CBCT technology)**	10 (66.67%) (8 public/2 private)	2 (13.33%) (1 public/1 private)	8 (53.33%) (6 public/2 private)	10 (66.67%) (9 public/1 private)	15 (12 public/3 private)
**Rotary or reciprocating endodontics**	10 (66.67%) (7 public/3 private)	9 (60%) (6 public/3 private)	13 (86.67%) (10 public/3 private)	12 (80%) (9 public/3 private)	15 (12 public/3 private)
**Chairside milling**	5 (33.33%) (3 public/2 private)	7 (46.67%) (6 public/1 private)	8 (53.33%) (6 public/2 private)	6 (40%) (5 public/1 private)	15 (13 public/2 private)
**Digitally designed surgical guides for implant placement**	5 (35.71%) (4 public/1 private)	1 (7.14%) (0 public/1 private)	10 (71.43%) (8 public/2 private)	4 (28.57%) (3 public/1 private)	14 (11 public/3 private)
**Intraoral scanning**	6 (46.15%) (4 public/2 private)	6 (46.15%) (5 public/1 private)	6 (46.15%) (5 public/1 private)	4 (30.77%) (4 public/0 private)	13 (11 public/2 private)
**Soft and/or hard tissue lasers**	5 (38.46%) (4 public/1 private)	2 (15.38%) (1 public/1 private)	9 (69.23%) (7 public/2 private)	5 (38.46%) (4 public/1 private)	13 (11 public/2 private)
**Extraoral scanning**	6 (50%) (5 public/1 private)	1 (8.33%) (1 public/0 private)	6 (50%) (5 public/1 private)	3 (25%) (3 public/0 private)	12 (10 public/2 private)
**Digital dentures (CAD/CAM CRDPs)**	5 (41.67%) (5 public/1 private)	0	7 (58.33%) (6 public/1 private)	2 (16.67%) (2 public/0 private)	12 (10 public/2 private)

* 1 private school reported the implementation of DDTs in their curriculum but failed to complete the questionnaire.

## Data Availability

The data presented in this study are available on request from the corresponding authors (H.A.A. and K.I.A.). The data are not publicly available due to privacy restrictions.
